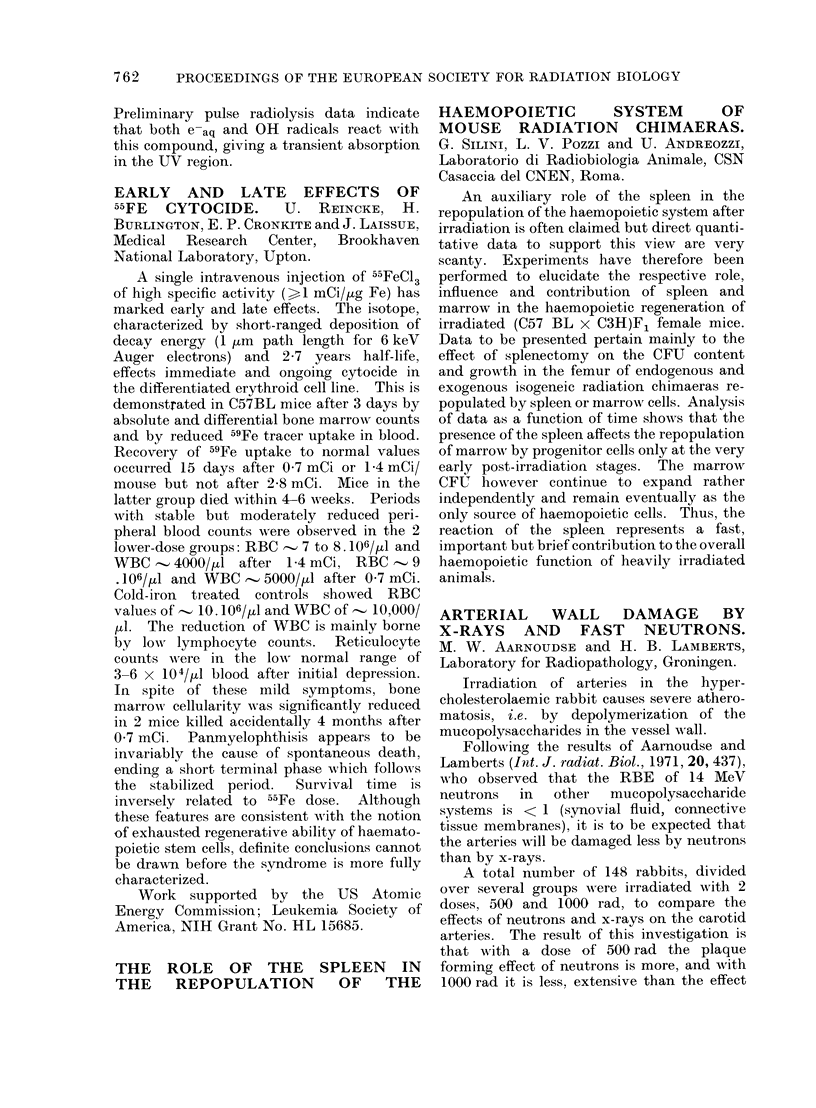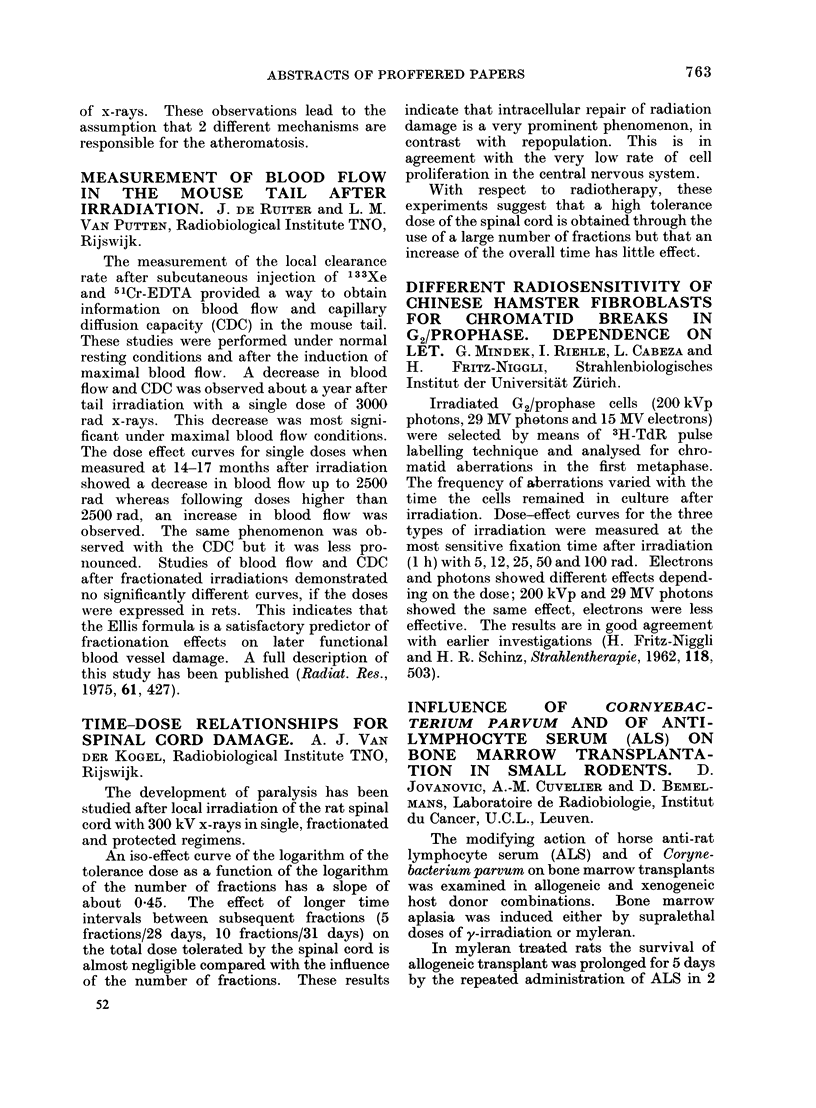# Proceedings: Arterial wall damage by x-rays and fast neutrons.

**DOI:** 10.1038/bjc.1975.327

**Published:** 1975-12

**Authors:** M. W. Aarnoudse, H. B. Lamberts


### ARTERIAL WALL DAMAGE BY X-RAYS AND FAST NEUTRONS.

M. W. Aarnoudse and H. B. Lamberts, Laboratory for Radiopathology, Groningen.

Irradiation of arteries in the hypercholesterolaemic rabbit causes severe atheromatosis, *i.e.* by depolymerization of the mucopolysaccharides in the vessel wall.

Following the results of Aarnoudse and Lamberts (*Int. J. radiat. Biol.*, 1971, **20**, 437), who observed that the RBE of 14 MeV neutrons in other mucopolysaccharide systems is $< 1$ (synovial fluid, connective tissue membranes), it is to be expected that the arteries will be damaged less by neutrons than by x-rays.

A total number of 148 rabbits, divided over several groups were irradiated with 2 doses, 500 and 1000 rad, to compare the effects of neutrons and x-rays on the carotid arteries. The result of this investigation is that with a dose of 500 rad the plaque forming effect of neutrons is more, and with 1000 rad it is less, extensive than the effect of x-rays. These observations lead to the assumption that 2 different mechanisms are responsible for the atheromatosis.